# HIF-2α Interaction with Ataxin-10 Enhances HIF-2α Binding to Its Target Gene Promoters

**DOI:** 10.3390/ijms262110417

**Published:** 2025-10-27

**Authors:** Aikaterini Diseri, Ioanna-Maria Gkotinakou, Christina Befani, Ioannis Pappas, Martina Samiotaki, George Panayotou, Panagiotis Liakos

**Affiliations:** 1 Laboratory of Biochemistry, Faculty of Medicine, University of Thessaly, Biopolis, 41500 Larissa, Greece; 2 Laboratory of Pharmacology and Toxicology, Faculty of Veterinary Science, School of Health Sciences, University of Thessaly, 43100 Karditsa, Greece; 3 Institute of Bioinnovation, BSRC “Alexander Fleming”, Vari, 16672 Athens, Greece

**Keywords:** hypoxia, HIF-2α, Ataxin-10, cervical cancer

## Abstract

The master transcription factors that control cell adaptation under hypoxia are known as hypoxia-inducible factors or HIFs. HIF-2α is the second isoform, which has been studied less extensively, and its expression is limited to particular cell types and is associated with increased malignancy in tumors. Herein, we investigate the interaction of HIF-2α with Ataxin-10, an intracellular protein involved in cell survival and differentiation, as well as the mechanism and the effects of this interaction in cervical cancer (HeLa) and glioma (U-87MG) cells. The interaction was investigated by LC-MS/MS proteomic analysis, immunoprecipitation, and immunoblotting. HIF-2 transcriptional activity was measured by luciferase assays and quantitative RT-PCR of target genes specific to HIF-2. The mechanism of interaction was investigated using immunofluorescence microscopy analysis, subcellular fractionation, siRNA-mediated silencing, quantitative RT-PCR, in vitro binding assays, and chromatin immunoprecipitation (ChIP). Ataxin interacts specifically with HIF2α and binds to the HIF-2α carboxyterminal activation domain. The interaction of HIF-2α with Ataxin-10 increases HIF-2-transcriptional activity under hypoxia through the enhancement of HIF-2α binding to chromatin in Hypoxia Response Elements of HIF-2 specific target genes *SERPINE1*, *CITED-2*, and *SOD-2*. These new data highlight a novel HIF-2 fine-tuning mechanism and may offer new, effective therapeutic approaches for treating cancerous tumors.

## 1. Introduction

Hypoxia is a prevalent characteristic of solid tumors and a crucial element of the tumor microenvironment, as it has a profound impact on various facets of tumor biology [1]. Major transcription factors that control the cell’s response to hypoxia are Hypoxia-inducible factors (HIFs). The HIF family consists of HIF-1 and HIF-2, which are heterodimers with a regulated alpha subunit and a stable beta subunit, also known as ARNT [2,3]. HIF-2α is very similar to HIF-1α, conserving 48 percent of its amino acid identity, mainly through structural and functional motifs. Despite this homology, HIF-2α shows a significantly different and distinct expression pattern, physiological role, regulatory control and specificity of the gene in oxygen homeostasis compared with HIF-1α [4]. Specifically, HIF-1α is characterized as ubiquitous, but HIF-2α is primarily expressed in the brain, heart, liver, lung, kidney, intestines, pancreas, and uterus [5,6,7,8,9]. In general, HIF-2α controls oxidative stress and vascular remodeling [10] and plays a role in metastasis, angiogenesis, and erythropoiesis [11,12,13,14]. Glioma cells express HIF-2α, whereas normal neural progenitors and glia do not [15,16], suggesting that HIF-2α is tumor-specific and may be clinically relevant as it is more prevalent in tumors and is associated with a higher grade of cancer and a lower chance of survival for patients [17]. HIF-2α inhibition has emerged as a novel therapeutic approach in clear-cell renal cell carcinoma (ccRCC) and other hypoxia-driven tumors. Belzutifan, a selective HIF-2α inhibitor, has shown significant clinical efficacy in ccRCC and von Hippel–Lindau-associated neoplasms. Emerging new inhibitors are under investigation and may further expand precision treatment options for hypoxia-related malignancies [18,19,20,21].

The stability of the α-subunit is negatively influenced by molecular oxygen concentration through hydroxylation reactions, which initiate the ubiquitin-proteasome degradation pathway [22,23,24]. HIF-2α stabilizes and moves to the nucleus as the amount of oxygen in the cell decreases, so that it can dimerize with ARNT. HIF-2α preferentially binds to the nucleus’s hypoxia response elements (HREs), which are of the 5′-RCGTG-3′ sequence of the DNA and the reverse sequence of the HRE, 5′-CACGY-3′, and is thereby involved in the transcriptional activation of these HIF-2 target genes [25].

Besides the required protein–protein interaction of HIF-2α with ARNT, a vast amount of different proteins is documented to bind with HIF-2α [21,26,27,28,29,30,31]. We have demonstrated in our recent findings an interaction between HIF-2α and Reptin52, which inhibits HIF-2 transcriptional activity [32]. Additionally, we have identified by mass spectrometry a new protein called Ataxin-10 that appears to bind with HIF-2α.

Ataxin-10 is a cytoplasmic protein encoded by the *ATXN10* gene. Although mutations or repeat expansions in *ATXN10* are associated with spinocerebellar ataxia type 10 (SCA10) [33,34,35], the molecular function of the protein remains poorly understood. Atxn10 knockout mice are embryonically lethal, indicating that Ataxin-10 is essential for normal development [36]. The protein contains two armadillo/HEAT repeat domains at its C-terminus and lacks transmembrane regions or other recognizable functional motifs [37]. Ataxin-10 has been reported to interact with the G-protein subunit β2 [38] and with O-linked β-N-acetylglucosamine transferase (OGT), linking it to processes such as neuronal development and maintenance of intracellular homeostasis through O-GlcNAcylation [39,40]. Furthermore, Ataxin-10 is phosphorylated by Aurora B and Polo-like kinase 1 (Plk1) at residues S77 and T82, which modulates its proteasome-dependent degradation and cytokinetic function [41,42]. These structural and regulatory characteristics suggest that Ataxin-10 may act as a scaffold protein facilitating interactions with other signaling molecules such as HIF-2α.

Herein, we report Ataxin-10 as a new HIF-2α-associated protein that contributes to its transcriptional activity. Specifically, we examined the effects of HIF-2α interaction with Ataxin-10 under hypoxia and the underlying mechanism of this interaction.

## 2. Results

### 2.1. HIF-2α Interacts with Ataxin-10

**Figure 1:**
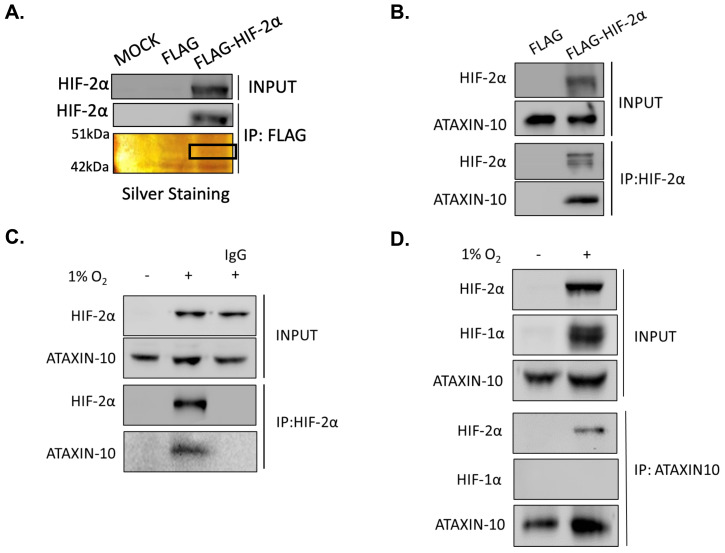
Ataxin-10 has been identified as a novel specific HIF-2α interaction partner. (**A**). SDS-PAGE HeLa extracts are used to analyze the proteins bound to wild-type HIF-2a by immunoblotting and by AgNO_3_ staining. The section of the gel analyzed by mass spectrometry is indicated in Black Square. (**B**). SDS-PAGE was used to analyze the immunoprecipitants, and immunoblotting against HIF2α and Ataxin-10 was carried out. (**C**). HeLa cells were cultured in a hypoxic (1 percent O_2_) environment for six hours. HIF-2α and Ataxin-10 antibodies were used for immunoblotting to examine total cell extracts (input) and HIF2α immunoprecipitated proteins (IP). (**D**). HeLa cells were cultured in a hypoxic (1 percent O_2_) environment for six hours. HIF-1α, HIF-2α and Ataxin-10 antibodies were used for immunoblotting to examine total cell extracts (input) and exposed to immunoprecipitation with an antibody against Ataxin-10. Immunoblotting was used to analyze immunoprecipitated proteins (IP) and total cell extracts (input), as indicated.

To gain insight into the regulatory mechanisms of HIF-2α, we investigated HIF-2α interaction with unknown proteins. After HIF-2α immunoprecipitation and AgNO_3_ staining of proteins, we distinguished a single 46 kDa protein identified by mass spectroscopy as Ataxin-10 (Figure 1A). The tryptic peptide VEQESLLTAFR of Ataxin-10 was identified with high confidence as a doubly charged ion (1292.68523) and with an XCorr of 3.18. To validate HIF-2α interaction with Ataxin-10, an anti-Flag antibody was used to immunoprecipitate HeLa cells that expressed Flag-tagged wild-type HIF-2α (Figure 1B). Next, to verify this interaction with endogenous HIF-2α, HeLa (Figure 1C) and U87MG (Figure S1A) cells were grown under hypoxia, and an anti-HIF-2α antibody was used to immunoprecipitate cells. According to the immunoprecipitant analysis, endogenous HIF-2α is linked to Ataxin-10. In the opposite experiment, Ataxin-10 immunoprecipitation from HeLa (Figure 1D), or U87MG (Figure S1B) cells further confirmed that endogenous HIF-2α interacts with Ataxin-10.

### 2.2. Under Hypoxic Conditions, Ataxin-10 Upregulates the Expression of HIF-2 Target Genes

**Figure 2:**
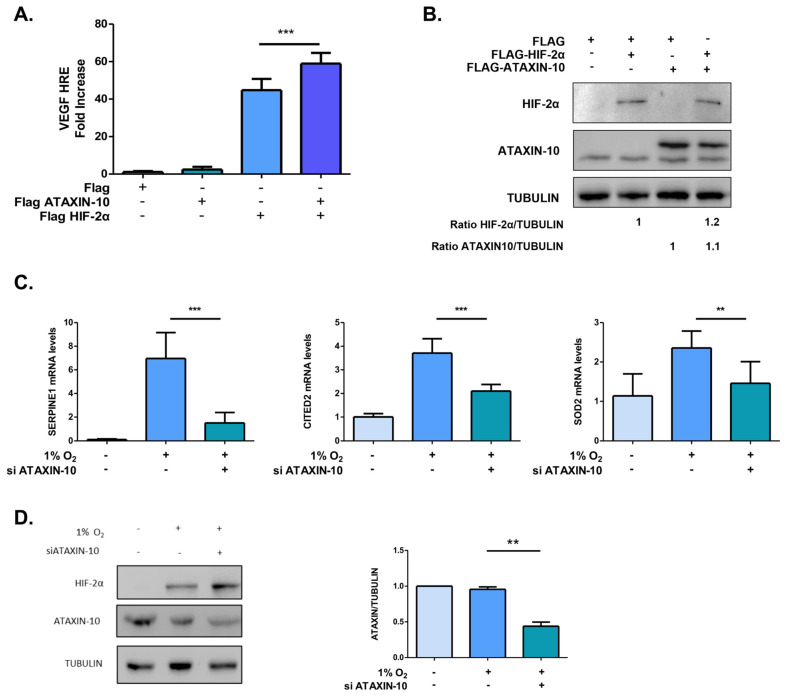
HIF-2 target gene expression is increased by Ataxin-10. (**A**) HIF-2 transcriptional activity following plasmid transfection of HeLa cells expressing HIF-2α, Ataxin-10 (or pFlag-CMV2 as a control), the firefly pGL3e5HRE-VEGF-luciferase and Renilla reporter plasmids and culturing in 21% O_2_ for 24 h. Values show the fold increase in relative luciferase units (Firefly over Renilla activity) in relation to the control and represent the mean of three independent experiments performed in triplicate ±SD (*** *p* < 0.001, comparisons were made by one-way ANOVA Tukey’s multiple comparisons). (**B**) HeLa cells were treated as in (**A**), and the lysates were tested against the designated epitopes with immunoblotting. (**C**) SERPINE1, CITED2, and SOD2 mRNA levels measured by RT-PCR in HeLa cells transfected with control siRNA or Ataxin-10-siRNA and incubated for 16 h at 1% O_2_. Results were shown as fold increase in relation to the corresponding normoxic conditions and represent the mean of two independent experiments performed in duplicate (±SD). (**D**) HeLa cells were transfected with control siRNA or Ataxin-10-siRNA and incubated for 16 h at 1% O_2_ similarly to (**C**), and HIF2α and Ataxin-10 protein were evaluated with immunoblotting (**left panel**). Quantification of protein levels of Ataxin-10 represents the average of three separate experiments ±SD (** *p* < 0.01, *** *p* < 0.001). One-way ANOVA was used for the comparisons. Multiple comparisons using Tukey’s test.

To determine if the association between HIF-2α and Ataxin-10 influences HIF-2 functions, a reporter assay utilizing *VEGF* HREs was employed to examine how overexpression of Ataxin-10 affects HIF-2 transcriptional activity. When compared to control, overexpression of Ataxin-10 increased HIF-2 activation by 50 times in HeLa cells and by 30 times in U87MG cells (Figure 2A,B; Figure S2A,B). Concurrent overexpression of Ataxin-10 increased HIF-2 transcriptional activity in both cell lines. In parallel, we evaluated the effect of Ataxin-10 silencing on HIF-2 transcriptional activity by measuring the mRNA levels of the HIF-2 specific target genes plasminogen activator inhibitor-1 (*SERPINE1*), superoxide dismutase 2 (*SOD2*) and Cbp/p300-interacting transactivator 2 (*CITED2*) in both cell lines by quantitative RT-PCR (Figure 2C,D; Figure S2C–F) [43,44,45,46,47,48,49,50,51,52,53]. The effect of Ataxin-10 silencing was also evaluated on the mRNA levels of HIF-1-specific target gene phosphoglycerate kinase 1 (PGK1) (Figure S1C). We observed that hypoxia significantly elevated *SERPINE1*, *SOD2*, and *CITED2* mRNA levels, whereas silencing Ataxin-10 significantly reduced their levels but not the HIF-1 target gene, *PGK1*. Taken together, these results suggest that Ataxin-10 strengthens only HIF-2 transcriptional activity under hypoxia.

### 2.3. Ataxin-10 Enhances HIF-2α’s Binding to Chromatin but Does Not Affect Its Subcellular Distribution

**Figure 3:**
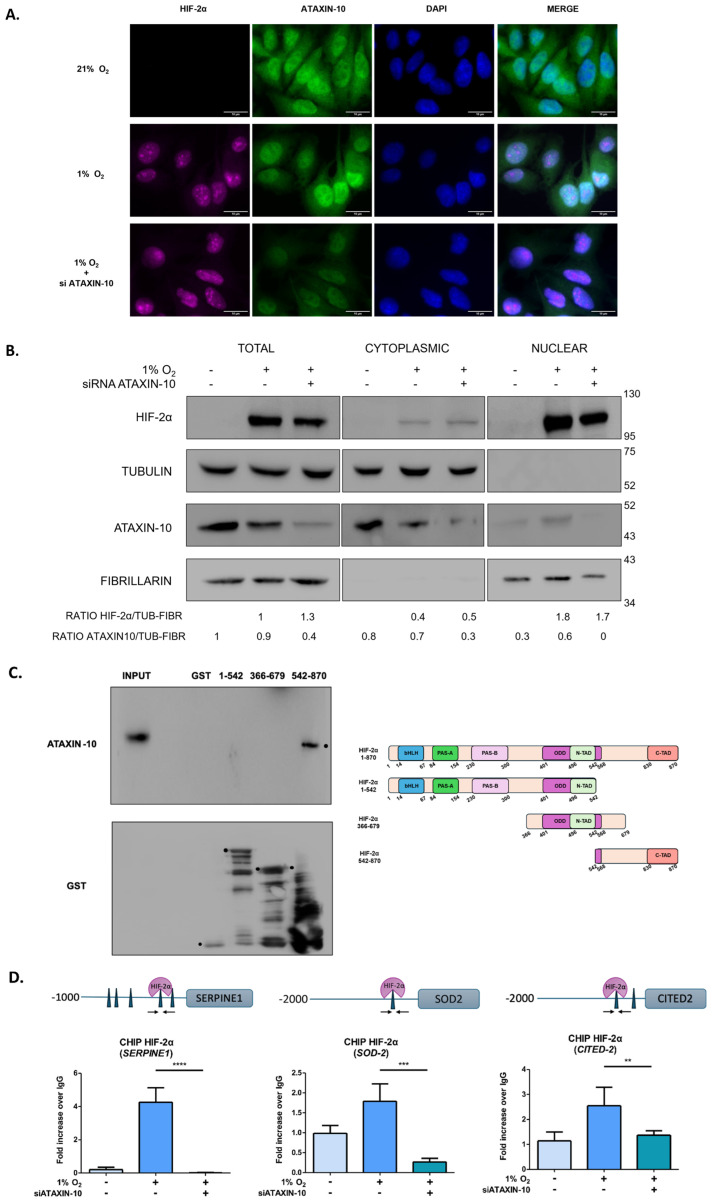
Ataxin-10 silencing decreases HIF-2 binding to the promoters of its target genes without changing the subcellular location of HIF-2α in hypoxic conditions. (**A**) An indirect immunofluorescence microscopy examination of HeLa cells treated with Ataxin-10 siRNA or control siRNA and incubated under normoxia or hypoxia for 16 h, using HIF-2α (LUT: Magenta) and Ataxin-10 (LUT: Green) antibody. DAPI (Blue) was used to stain the nuclei. Blue, green, and magenta LUTs are combined to create Merge (scale bars: 10 μm). (**B**) Immunoblotting of HeLa cell nuclear, cytoplasmic, or total extracts, as in (**A**), using antibodies against HIF-2α and Ataxin-10. Tubulin and fibrillarin served as cytoplasmic or nuclear markers, respectively. The position of MW is displayed on the left. Ratio HIF-2a or Ataxin-10/tubulin or fibrillarin is expressed as fold change relative to control. (**C**) GSH-Sepharose beads containing GST or GST-HIF-2α fragments (as specified) were mixed with HeLa lysates, and 2x SDS was used for elution. Antibodies against Ataxin10 and GST were used in immunoblotting (WB) to examine the eluted proteins (**left panel**). Diagrammatic depiction of the domain structure of either the truncated or full-length GST-tagged HIF-2a forms used in vitro binding (**right panel**). MW’s location is displayed on the left. (**D**) Upper panel: Schematic representation of SERPINE1, CITED2, and SOD2 promoter regions indicating the potential HRE sequences (Triangles). Arrows indicate the set of primers for DNA amplification after ChIP. The presence of HIF-2 (Purple Circle) indicates the HRE sequences, which are located at its peak, identified from the public ChIP data. Lower panel: HeLa cells treated as shown in (**A**) were used to produce qRT-PCR results from anti-HIF-2α or rabbit IgG chromatin immunoprecipitants using primers specific for the HRE of the SERPINE1, SOD-2, and CITED-2 promoter. The values represent the mean of two separate experiments conducted in triplicate ±SD and are presented as a fold increase in comparison to IgG ChIP (** *p* < 0.01, *** *p* < 0.001, **** *p* < 0.0001). Tukey’s multiple comparisons and one-way ANOVA were used for comparisons.

To investigate whether Ataxin-10 influences the subcellular localization of HIF-2α, both endogenous proteins were investigated in normoxic and hypoxic HeLa (Figure 3A) and U87MG (Figure S3) cells by immunofluorescence microscopy. A little portion of hypoxia-induced HIF-2α was detectable in the cytoplasm, while the majority was nuclear, both under hypoxia (1% O_2_) and under hypoxia with Ataxin-10 siRNA treatment (Figure 3A). In U87MG cells, a comparable pattern was seen, with HIF-2α remaining primarily nuclear under hypoxia both with and without ataxin-10 (Figure S3). Under normoxia, Ataxin-10 was detected both in the nucleus and in the cytoplasm. Under hypoxia, a larger portion of Ataxin-10 was detected in the nucleus (Figure 3A). To confirm these results, we performed biochemical fractionation after silencing Ataxin-10 in HeLa cells (Figure 3B). Both HIF-2α and Ataxin-10 were found in the cytoplasmic and nuclear fractions, which is consistent with immunofluorescence results. Upon Ataxin-10 silencing, there was no change in HIF-2α subcellular distribution, indicating that there are no notable changes in the subcellular distribution of HIF-2α associated with the impact of Ataxin-10 on HIF-2 activation. The consequence of Ataxin-10 silencing on the half-life of HIF-2α was examined following cycloheximide treatment in HeLa cells (Figure S4A). When comparing cells with silenced Ataxin-10 to those under hypoxia, we found that the stability of the HIF-2α protein remained unchanged. Furthermore, RT-PCR was used to measure the HIF-2α mRNA levels in HeLa cells when Ataxin-10 was silenced, and it was found that Ataxin-10 does not affect HIF-2α mRNA levels (Figure S4B).

To identify the HIF-2α region that is required for its interaction with Ataxin10, we have performed additional experiments. Recombinant fragments corresponding to amino acids 1–542, including the HIF2α bHLH-PAS, the ODD and the N-TAD domains, 366–679, including the ODD, the N-TAD and part of the Inhibitory (IH) domain, and 542–870, including part of ODD, IH domain and the C-TAD half responsible of transcriptional activity domain, were used as baits in pull-down assays with HeLa cell extracts. The results showed a strong affinity of Ataxin10 with the 542–870 GST-HIF-2α form but not for 366–679 GST-HIF-2α form. Taking these results into account, we conclude that the strong association of HIF-2α with Ataxin10 involves amino acids 679–870 with part of IH domain and the C-TAD region of HIF-2α, which is important for its transcriptional activation (Figure 3C). Finally, we used an anti-HIF-2α antibody to do chromatin immunoprecipitation assays, and then we used RT-PCR analysis on gene promoters that contained well-characterized HIF-2 hypoxia-responsive elements such as *SERPINE1*, *SOD-2*, and *CITED-2*. Depletion of Ataxin-10 considerably decreased HIF-2’s binding to the target genes’ HRE promoter, according to analysis (Figure 3D). Our findings showed that Ataxin-10, by promoting a robust connection of HIF-2α with the promoters of its target genes, activates HIF-2.

## 3. Discussion

The tumor microenvironment is characterized by hypoxia, which is connected to both tumor aggression and treatment resistance. These disorders result in elevated HIF-2α transcriptional activity, which has a direct impact on stemness, angiogenesis, and metastasis [10,18,54]. Thus, it is fundamental to investigate the molecular processes that regulate HIF within cells. HIF-2α is located mainly in the nucleus at predetermined sites with a finite size and capacity, and to a lesser extent, in the cytoplasm. Here, we revealed that Ataxin-10 is a new partner of the HIF-2α protein. Specifically, we have shown that endogenous and overexpressed HIF-2α interact with Ataxin-10 in cervical and glioblastoma cells. This interaction is specific for HIF-2α, as Ataxin-10 and HIF-1α do not interact (Figure 1D). Additionally, we noticed that this interaction results in increased HIF-2 transcriptional activity. By examining the interaction between HIF-2α and Ataxin-10, we demonstrated that Ataxin-10 binds to amino acids 679–870, including part of the inhibitory domain and the C-TAD domain of HIF-2α, which is important for its transcriptional activation (Figure 3C). Future studies should aim to characterize the molecular details of the Ataxin-10/HIF-2α interaction, for example through protein–protein modeling, mutagenesis of the armadillo/HEAT domains, or in vitro binding assays, to elucidate the structural basis and regulation of this interaction.

Moreover, the HIF-2α/Ataxin-10 interaction increases the mRNA levels of HIF-2 target genes *SERPINE*, *SOD-2*, *CITED-2* but not HIF-1 target gene, and Phosphoglycerate Kinase 1 (*PGK-1*) (Figure S1C), supporting the result that Ataxin-10 specifically interacts with HIF-2α. Ataxin-10 is predominantly localized in the cytoplasm and perinuclearly [41]. Here, we demonstrate that hypoxia shifts the balance in favor of Ataxin-10 localization in the nucleus. Our results indicate that Ataxin-10 depletion in hypoxia affects the mRNA levels of the HIF-2 target gene without altering sub-cellular distribution of HIF-2α, indicating that the shift in Ataxin-10 localization in the nucleus contributes to enhancing HIF-2 activity.

In addition, we have found that under hypoxic conditions, Ataxin-10 enhances HIF-2’s adherence to HRE in the target gene’s promoter and contributes to making nuclear HIF-2 fully transcriptionally active. Ataxin-10 functions are largely unknown and are mainly associated with the cytoplasm in 21% O_2_. In this study, we have shown for the first time a new role of Ataxin-10 under hypoxia (1%). Ataxin-10 was found to function as a cofactor for the full transcriptional activation of HIF-2 and appears to be another member of the increasing list of factors that cooperate with HIF-2 for its highest and most specific transcriptional upregulation. Actually, it is well known that HIF-2α interacts with several members of the ETS family, including ETS-1 and ELK, that may contribute to target gene selection [29,30]. Also, HIF-2α forms a transcriptional complex with USF2, RNA polymerase II, and CBP/p300 to promote the transcription of HIF-2 target genes under hypoxic conditions [26,27,29]. In comparison to other well-characterized HIF-2α co-factors, which enhance HIF-2α–dependent gene expression by directly promoting its transcriptional activation, Ataxin-10 appears to act through a distinct mechanism, primarily facilitating HIF-2α binding to target gene promoters rather than modulating its intrinsic transactivation potential. However, future studies using ATAC-seq or histone mark profiling will be important to determine whether Ataxin-10 regulates chromatin engagement by enhancing HIF-2α stability at active promoters or by recruiting transcriptional cofactors. In particular, investigating potential interactions between Ataxin-10 and cofactors such as CBP/p300 could clarify its role in facilitating HIF-2α–mediated gene activation. Our current results demonstrate that Ataxin-10 enhances HIF-2α transcriptional activity, promoting the expression of HIF-2α–target genes. This finding contrasts with our previous report on Reptin52, which interacts with HIF-2α to suppress its transcriptional function [32]. These contrasting effects suggest that HIF-2α activity is modulated by a dynamic balance between activating and repressive cofactors.

**Figure 4:**
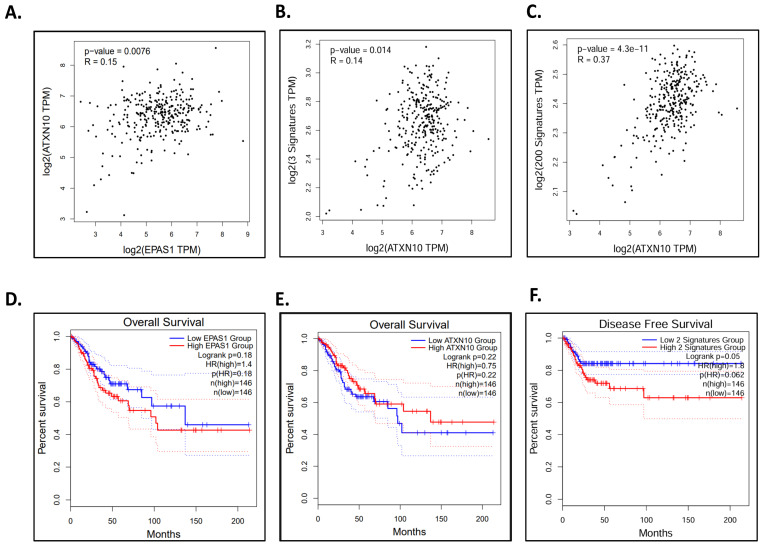
Correlation and Survival analysis between EPAS1 and ATXN10 with patient data. (**A**–**C**) Pearson correlation analysis between ATXN10 (Ataxin-10) and EPAS1 (HIF-2α) levels using TCGA-CESC RNA-seq data (n = 306), *p* < 0.05 was considered significant. (**A**) Association between HIF-2α and Ataxin-10 expression in patient samples with cervical squamous cell carcinoma and endocervical adenocarcinoma (TCGA-CESC) tumors using the Gepia2 web database. (**B**) Association between expression of Ataxin-10 and SERPINE1, SOD2, and CITED2 in cervical cancer patient samples using the Gepia2 web database. (**C**) Association between expression of Ataxin-10 and signature genes of hypoxia in cervical cancer patient samples using the Gepia2 web database. (**D**–**F**) Kaplan–Meier overall and disease-free survival curves for EPAS1 and ATXN10 expression. Patients were divided by median expression (50% high vs. 50% low; n high = 146, n low = 146). The hazard ratio (HR) for EPAS1 high expression was 1.4 (*p* = 0.18; log-rank test), while for ATXN10 it was 0.75 (*p* = 0.22; log-rank test). HRs, 95% confidence intervals (CIs), and *p*-values are indicated in each panel. (**D**) Kaplan–Meier overall survival curves for patients with cervical cancer based on whether they express HIF-2α at high (Red) or low (Blue) levels. (**E**) Kaplan–Meier overall survival curves for Ataxin-10 at high (Red) or low (Blue) levels in tumor samples, and (**F**) Kaplan–Meier Disease Free Survival between HIF-2α and Ataxin-10 expression in tumor patient samples using the Gepia2 web database (log-rank *p*-values shown in each panel). Solid lines are the mean values from the data. The 95% confidence interval is plotted by the dotted lines.

These results indicate that HIF-2 requires Ataxin-10 to be fully active. The analysis of publicly available cervical cancer patient data also supports our results. The publicly available glioma cancer patient data are limited and do not allow statistically significant data to be extracted. It was observed that in cervical cancer patient samples with elevated HIF-2α increased expression of Ataxin-10 occurs with a *p*-value of 0.0076 (Figure 4A). The disease-free survival of patients was identified, and a statistically significant association was observed between low expression of HIF-2α and Ataxin-10 and patient survival (*p* = 0.05) (Figure 4). In addition, the analysis shows a significant correlation of Ataxin-10 mRNA levels and HIF-2 target genes (*p* = 0.014) in cervical cancer patient samples from the Gepia2 database (presented in Figure 4B). Likewise, we examined the correlation between Ataxin-10 expression and the signature genes of hypoxia, and we observed a statistical correlation (*p* = 4.3 × 10^−11^) (Figure 4C). These findings show that elevated expression of HIF-2α and Ataxin-10 is linked to a poor prognosis for cervical cancer (Figure 4F). These results show that the increased expression of HIF-2α and Ataxin-10 is associated with poor prognosis in cervical cancer, and their expression correlation fully supports our in vitro cell data. A limitation of this study is that the clinical relevance of *ATXN10* and *EPAS1* expression was evaluated based on publicly available transcriptomic datasets (GEPIA2/TCGA-CESC). Although these analyses provide valuable insights, they rely solely on in silico correlations. Future studies including immunohistochemical or protein-level validation in patient-derived cervical cancer samples will be essential to confirm the observed associations.

In conclusion, our study highlights a novel protein interaction of HIF-2α that increases its transcriptional activity. Also, this interaction affects its binding to target gene promoters. Under hypoxia, Ataxin-10 acts as a HIF-2 coactivator that regulates HIF-2-dependent transcription and function. These new data provide new knowledge on the activity of HIF-2 and may offer new effective therapeutic approaches to treat cancerous tumors.

## 4. Materials and Methods

### 4.1. Cell Culture, Transfection, and Luciferase Assays

Both the HeLa (CVCL_R965) human cervical carcinoma and the human glioblastoma U87-MG (CVCL_0022) cell lines were obtained from the European Collection of Cell Cultures (ECACC, Salisbury, UK) and examined for mycoplasma contamination. For the purpose of hypoxic treatment, an INVIVO_2_ 200 hypoxia workstation (Baker Ruskinn, Sanford, ME, USA) was used to cultivate cells at 1% O_2_, 94%N_2_, and 5% CO_2_. Cell culture, hypoxic conditions, and transient transformation experiments were performed according to Gkotinakou IM et al., 2021 [32]. To inhibit protein biosynthesis, cells were treated with Cycloheximide (CHX) (C4859, Merck, Darmstadt, Germany) and incubated under hypoxia. To perform luciferase assays, cells were co-transfected with plasmids that expressed HIF-2α, Ataxin10 (or pFlag-CMV2 as a control; Dr. Jing Li [41] kindly provided the Ataxin-10 plasmid), and the reporter plasmids PCI-Renilla-Luciferase and pGL3e5HRE-VEGF firefly-luciferase. Luciferase activity was measured 24 h post-transfection using the Dual-Luciferase Reporter Assay System (Promega, Madison, WI, USA) and Spark^®^ multi-mode plate reader (Tecan, Zurich, Switzerland).

### 4.2. siRNA-Mediated Silencing, RNA Extraction, cDNA Production, and Quantitative PCR

Using Lipofectamin2000 (11668019, Invitrogen, Waltham, MA, USA), HeLa and U87MG cells received treatment with 20 nM siRNA targeting Ataxin10 (sc-60218, Santa-Cruz Biotechnology, Dallas, TX, USA) in compliance with the manufacturer’s instructions. Ataxin-10 siRNA (human) consists of a pool of three target-specific 19–25 nt siRNAs designed to knock down gene expression. AllStars siRNA (1027280, Qiagen, Venlo, The Netherlands) was employed as a negative control. 24 h after transfection, cells were subjected to 16 h of 1% O_2_. Following cell collection, lysates were prepared for RT-PCR or Western blotting. Nucleozol (Macherey Nagel, Düren, Germany) was used to extract total RNA from cells for RT-PCR, and cDNA was produced using the High-Capacity Reverse Transcription kit (Thermo Fisher Scientific, Waltham, MA, USA). Using primers for *SERPINE1*: FOR 5′-CAGCTGACAACAGGAGGAGA-3′, REV 5′-CCCATGAGCTCCTTGTACAGAT-3′, *CITED2*: FOR 5′-CCTACCCCCACAACCACTACA-3′, REV 5′-GCAATCTCGGAAGTGCTGGT-3′, *SOD2*: FOR 5′-GCACTAGCAGCATGTTGAGC-3′, REV 5′-CTCCTCGGTGACGTTCAGG-3′, *18S*: FOR 5′-CTCAACACGGGAAACCTCAC-3′, REV 5′-CGCTCCACCAACTAAGAACG-3′, qPCR was performed using SYBR^®^Green (Thermo Fisher Scientific, MA, USA) in a LightCycler 96 instrument (Roche Life Science, Basel, Switzerland). Samples for the internal control and the target were tested twice. The relative mRNA expression was determined using the DDCT method and presented as a fold increase relative to the respective controls.

### 4.3. LC-MS/MS Proteomic Analysis

Proteomic analysis was performed using LC–MS/MS as previously described [55]. Briefly, protein samples underwent in-gel tryptic digestion, and the resulting peptides were extracted, dried by vacuum centrifugation, and reconstituted in 2% acetonitrile containing 0.1% formic acid. Peptide analysis was conducted on an LTQ Orbitrap XL mass spectrometer (Thermo Fisher Scientific, Waltham, MA, USA) coupled to a nano-HPLC system using a reversed-phase C18 column. Full-scan MS spectra were acquired in the Orbitrap at a resolution of 60,000, followed by data-dependent MS/MS fragmentation of the six most intense ions using collision-induced dissociation (CID). Instrument control and data acquisition were performed using Xcalibur software version 4.7.69 (Thermo Fisher Scientific), and peptide identification was carried out in Proteome Discoverer v1.4 using the SEQUEST HT algorithm and the *UniProt* human FASTA database (October 2015). Search parameters included strict trypsin specificity (up to two missed cleavages) and variable modifications of methionine oxidation, deamidation (N/Q), and N-terminal acetylation.

Detailed chromatographic parameters (column specifications, gradient profile, flow rate, and temperature settings) are provided in the Supplementary Materials.

### 4.4. Immunoprecipitation

Antibodies against HIF-2α (NB100-122, Novus Biologicals LLC, Centennial, CO, USA) and Ataxin-10 (sc-271233, Santa-Cruz Biotechnology, TX, USA) were used to immunoprecipitate proteins from whole cell extracts, rotated for 16 h at 4 °C in a buffer (10 mM Tris-HCl pH 7.4, 150 mM NaCl, 2 mM EDTA, 1 mM EGTA, 1% Triton X-100, 1 mM NaVO_3_, 10 mM NaF, 10mM β-glycerophosphate and PI MIX). 2x Laemmli buffer was used to elute the precipitated proteins, which were then resolved in 10% SDS-PAGE and examined by immunoblotting with the appropriate antibodies.

### 4.5. Subcellular Fractionation

HeLa cells treated with siRNA targeting Ataxin-10 and cultivated for 16 h under hypoxia were collected using Nucleoli standard buffer (10 mM Tris-HCl, pH 7.4, 10 mM NaCl, 1 mM MgCl_2_) and incubated on ice for 30 min. Then, for homogenization, NP40 was added together with the cell suspension in a Dounce. A solution of 10 mM MgCl_2_ and 250 mM sucrose was used to resuspend the pellet, and under the suspension buffer was added containing 880 mM of sucrose and 5 mM of MgCl_2_. The pellet, which represents the nuclear fraction, was obtained after centrifuging the solution for 10 min at 1200× *g*. Following that, SDS-PAGE and immunoblotting using the appropriate antibodies were used to evaluate the fractions.

### 4.6. Western Blot Analysis and Immunofluorescence Microscopy

Proteins were separated in 10% SDS-PAGE, and then rabbit monoclonal antibodies against HIF-2α (59973, Cell Signaling Technology, Danvers, MA, USA) and Fibrillarin (C13C3, Cell Signaling Technology, MA, USA), as well as mouse monoclonal antibodies against Ataxin-10 (sc-271233, Santa-Cruz Biotechnology, TX, USA), a-Tubulin (3873, Cell Signaling Technology, MA, USA), and Flag (F4042, Sigma Aldrich, St. Louis, MO, USA), were used for immunoblotting analysis. A Uvitec Cambridge Chemiluminescence Imaging System with Alliance Software (version 16.06) was used to expose immunoblots. In order to perform immunofluorescence microscopy, cells cultured on coverslips were permeabilized at 4 °C with 0.1% Triton-X 100, fixed with 3% formaldehyde/PBS 1x for 5 min, and incubated for 1 h with BSA at room temperature. After that, they were treated with primary antibodies diluted in blocking solution for a whole night at 4 °C and followed by incubation with secondary antibodies conjugated with Alexa 488 and Alexa 647 at room temperature for one hour. Images were collected with a Zeiss Axio Imager Z2 microscope equipped with an AxioCamMRm sensor (Zeiss (Carl Zeiss AG), Oberkochen, Germany) and a 100× oil immersion lens. Analysis of the images was performed on ImageJ (Fiji v1.52p).

### 4.7. In Vitro Binding Assays

The in vitro binding tests were carried out as previously mentioned [32]. Briefly, 0.5 mL of HeLa cell extracts (~1 mg protein) were treated with 5 mg of GST or GST-HIF-2α forms immobilized on 15 μL glutathione-Sepharose beads for one hour at 4 °C. Proteins attached to GST-HIF-2α forms were eluted using 2x Laemmli buffer following bead washing and analyzed with Western Immunoblotting with proper antibodies.

### 4.8. Chromatin Immunoprecipitation (ChIP)

Cells after treatment with siRNA targeting Ataxin-10 and 16 h under hypoxia were exposed to 1.1% formaldehyde for 30 min at room temperature, quenched for 5 min with 125 mM of glycine, and then washed with three buffers at 4 °C: (a) PBS 1x, (b) 0.25% Triton X-100, 0.5 mM EGTA, 10 mM EDTA, 20 mM HEPES, pH 8, and (c) 0.15 M NaCl, 0.5 mM EGTA, 1 mM EDTA, 20 mM HEPES pH 8. The cells were subsequently sheared using a Fisherbrand^TM^ Model 50 Sonic Disembranator (Thermo Fisher Scientific, Waltham, MA, USA) after being suspended in ChIP incubation buffer (1% Triton X-100, 0.15% SDS, 150 mM NaCl, 0.5 mM EGTA, 1 mM EDTA, and 20 mM HEPES, pH 8). Following a 5-min centrifugation, the sonicated chromatin was treated overnight with purified rabbit HIF-2α antibody and appropriate IgG antibody using protein A/G beads. The beads were cleaned six times at 4 °C using various buffers: twice with a solution of 0.1% SDS, 0.1% DOC, 1% Triton, 1 mM EDTA, 150 mM NaCl, 0.5 mM EGTA, 20 mM HEPES, pH 8, once with the same solution but with 500 mM of NaCl, once with a solution of 0.25 M LiCl, 0.5% NP-40, 0.5% DOC, 0.5 mM EGTA, 1 mM EDTA, 20 mM HEPES, pH 8, and twice with, 0.5 mM EGTA, 1 mM EDTA and 20 mM HEPES, pH 8. 400 μL of elution buffer (1% SDS, 0.1 M Na-HCO_3_) was used to elute the precipitated chromatin. It was then incubated at 65 °C for five hours with 0.2 M NaCl, phenol was extracted, and it was precipitated with 2.5 V 100% ethanol and 1/10 V 1 M CH3COOK at −20 °C overnight. The regions of the SERPINE1, SOD-2, and CITED-2 promoters, containing known, well-characterized and functional HREs, identified using HeLa Chip data [13,43,50,53], and the following primers were used to amplify them from immunoprecipitated chromatin or input samples: *SERPINE1* FOR 5′-TTGAGCCCAGGAGTTTGAG-3′, REV 3′-CTCCCAAGTAGCTGGGAATAC-5′, *SOD-2* FOR 5′-TCCCAGGTTCAAGCAATTCTC-3′, REV 5′-TGGAGAAACCCTGTCTCTACTAA-3′, *CITED-2* FOR5′-TTCCAGTCCGCGCTTTC-3′, REV 5′-CGGATGCCAAAGCTACCAAG-3′. The products were quantified using real-time qPCR. The threshold cycle (Ct) of samples that were immunoprecipitated using either specific or non-specific IgG antibodies was compared to the threshold cycle of the total input DNA (ΔCt). After normalizing the antibody results to those obtained with non-specific IgG (ΔΔCt), the fold increase compared to the non-specific IgG condition was reported.

### 4.9. Statistical Analysis

Two sets of data were compared statistically using a one-way ANOVA. GraphPad Prism 5.04 was used to perform Tukey’s multiple comparison analysis; *p* < 0.05 was deemed significant (* *p* < 0.05; ** *p* < 0.01; *** *p* < 0.001).

## Data Availability

The original contributions presented in this study are included in the article/Supplementary Materials. Further inquiries can be directed to the corresponding author.

## Supplementary Materials

The supporting information can be downloaded at: https://www.mdpi.com/article/10.3390/ijms262110417/s1.
