# Supplementary material for: HIF-2α Interaction with Ataxin-10 Enhances HIF-2α Binding to Its Target Gene Promoters
Source: Int J Mol Sci. 2025 Oct 27;26(21):10417. doi: 10.3390/ijms262110417 (PMC12607750; doi:10.3390/ijms262110417)
Supplement: Supplementary file 1 [file ijms-26-10417-s001.zip › ijms-3929689-supplementary.pdf]

Supplementary Material

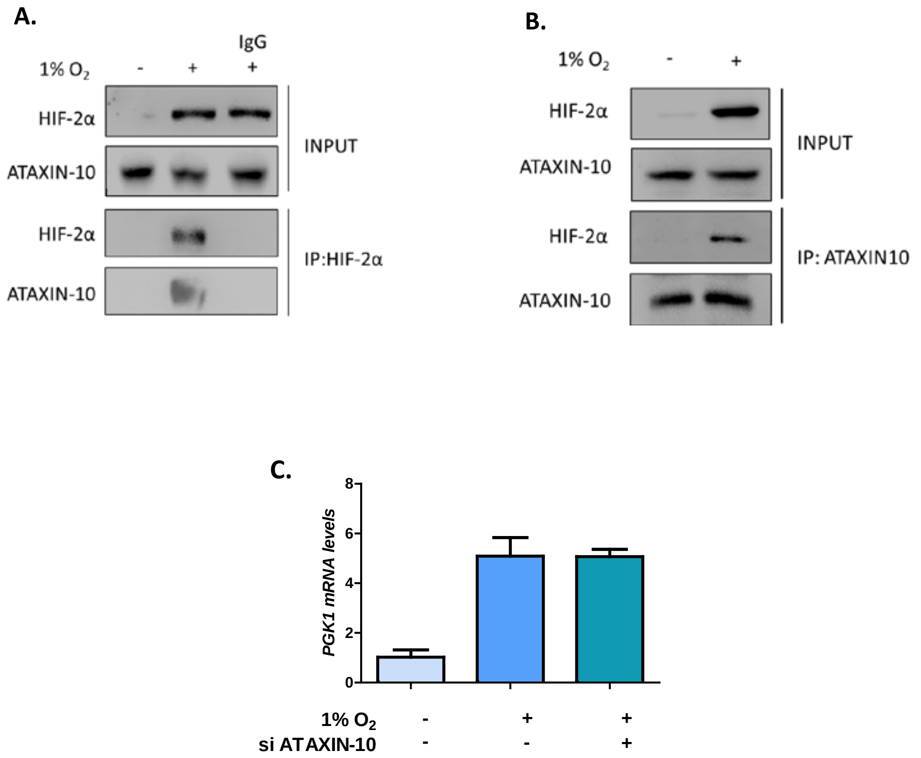

Figure S1: Identification of Ataxin-10 as a new interaction partner of HIF-2α. A) U87MG cells were incubated for 6 h under hypoxia (1%O2). Total cell extracts (input) and HIF2α immunoprecipitated proteins (IP) were analyzed by immunoblotting using HIF-2α and Ataxin-10 antibodies. **B)** U87MG cells were treated as in A. and subjected to immunoprecipitation using anti-Ataxin-10 antibody. Total cell extracts (input) and immunoprecipitated proteins (IP) were analyzed by immunoblotting as indicated. **C)** Phosphoglycerate kinase 1 (PGK-1) mRNA levels measured by RT-PCR in HeLa cells transfected with control siRNA or Ataxin-10-siRNA and kept under 1% O_2. Results are shown as fold increase in relation to the corresponding normoxic conditions and represent the mean of three independent experiments performed in duplicate (±SD).

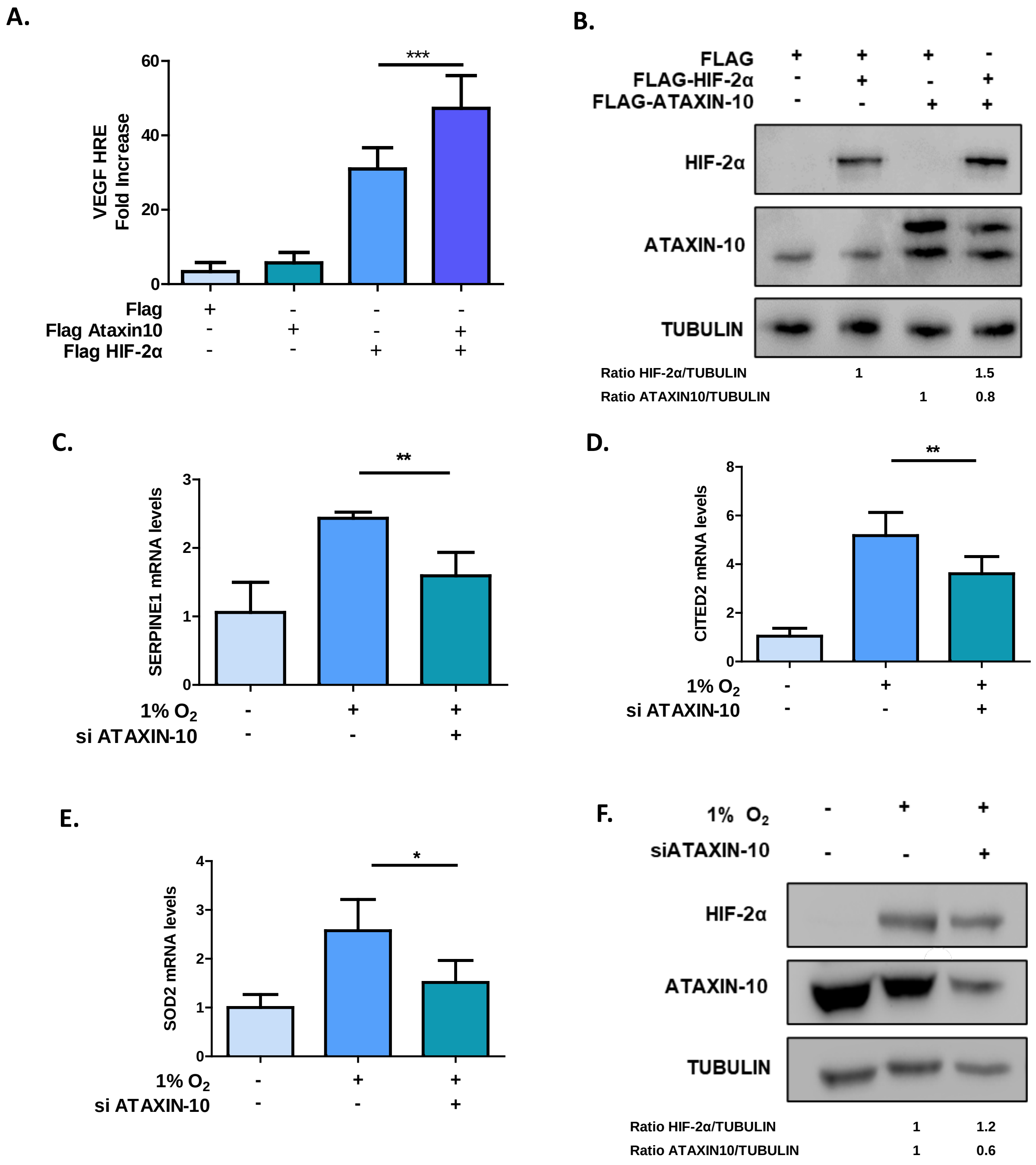

Figure S2: Ataxin-10 upregulates the expression of HIF-2 target genes. HIF-2 transcriptional activity after transfecting A) U87MG cells with plasmids expressing HIF-2α, Ataxin-10 (or pFlag-CMV2 as control), and the firefly pGL3e5HRE-VEGF and Renilla reporter plasmids. Values show the fold increase in relative luciferase units (Firefly over Renilla activity) in relation to the control and represent the mean of three independent experiments performed in triplicate ±SD (***$p < 0.001$, comparisons were made by one-way ANOVA Tukey's multiple comparisons). **B)** U87MG cells transfected with plasmids expressing HIF-2α, Ataxin-10, or pFlag-CMV2 as a control, the cells were cultured in 21%O2 for 24 hours. Immunoblotting was used to evaluate the lysates against the designated epitopes. C) SERPINE1, D) CITED2 and E) SOD2 mRNA levels measured by RT-PCR in U87MG cells transfected with control siRNA or Ataxin-10-siRNA and kept under 1%O_2 for 16 hours. Results are shown as fold increase in relation to the corresponding normoxic conditions and represent the mean of two independent experiments performed in duplicate (±SD). **F)** U87MG cells transfected with either Ataxin-10-siRNA or control siRNA, then incubated for 16 hours at 1% oxygen. Immunoblotting was used to evaluate the lysates against the designated epitopes.

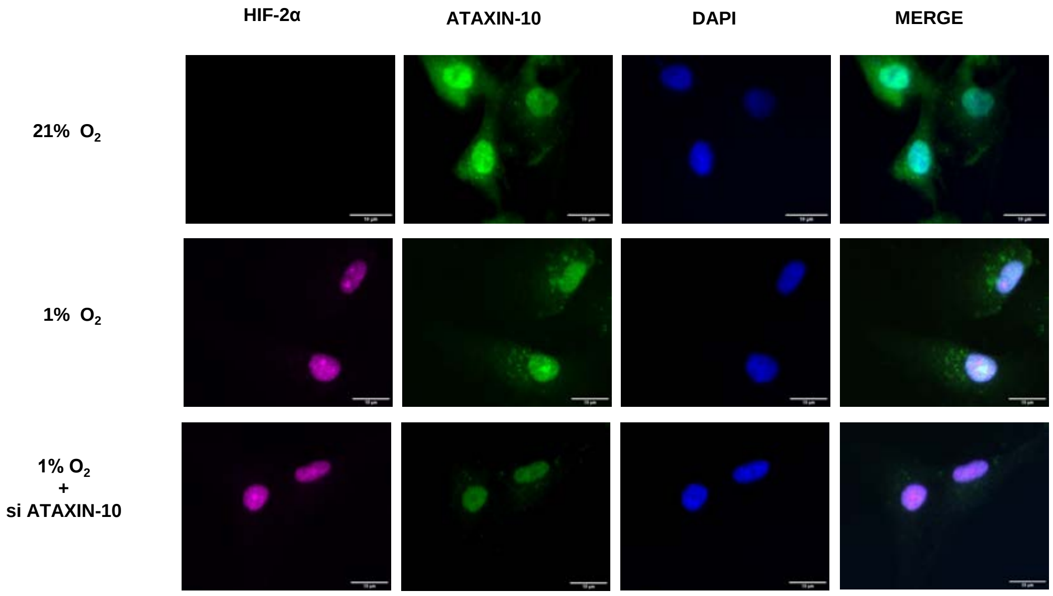

Figure S3: Knockdown of Ataxin-10 does not affect HIF-2α localization. Using HIF-2α (LUT: Magenta) and Ataxin-10 (LUT: Green) antibodies, indirect immunofluorescence microscopy examination was performed on U87MG cells transfected with siRNA for Ataxin-10 or control siRNA and cultured under normoxia or hypoxia for 16 hours. DAPI (Blue) staining was done on the nuclei. The combination of blue, green, and magenta LUTs is called a merge (scale bars: 10 mM).

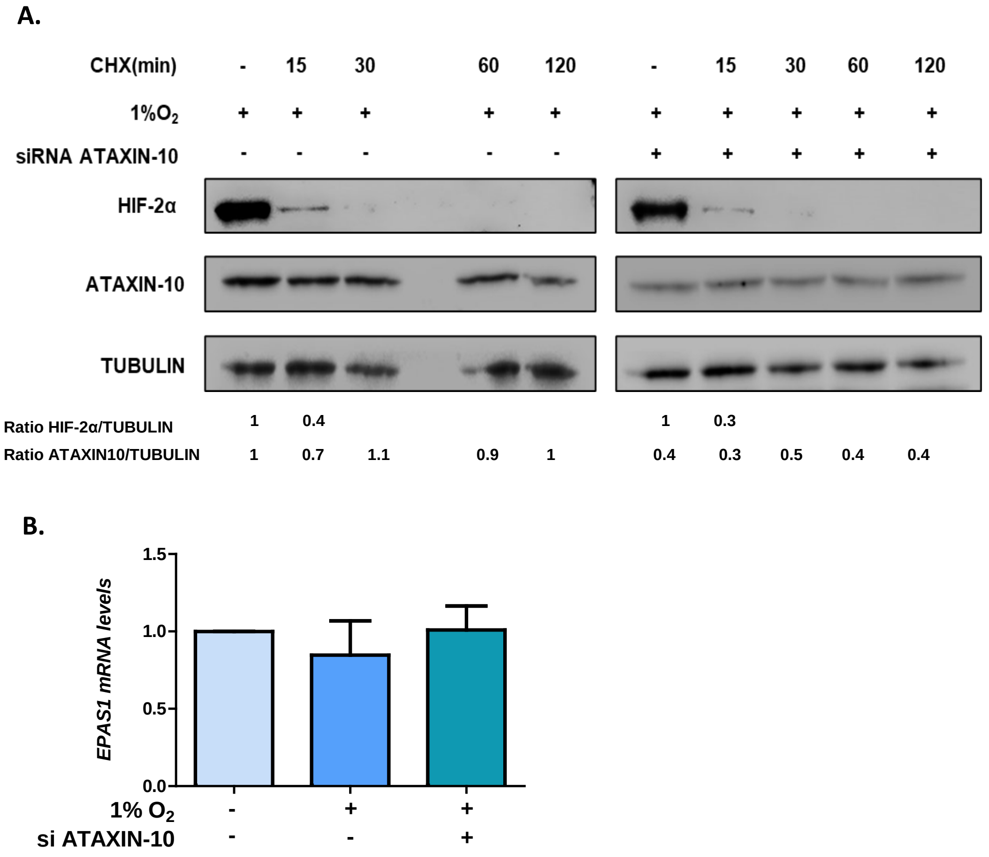

Figure S4: Depletion of Ataxin-10 does not affect HIF2α half-life and mRNA levels. A) HeLa cells were treated with control siRNA or Ataxin-10-siRNA. After 24 hours after transfection, the cells were treated with CHX (25 mg/ml) for the prescribed amount of time after being incubated in 1%O2 for 6 hours. Immunoblotting was used to evaluate the lysates against the designated epitopes. **B)** RT-PCR was used to determine the RNA levels of EPAS1 (HIF-2α) in HeLa cells transfected with either control or Ataxin-10-siRNA and maintained at 1% oxygen. The findings are expressed as a fold increase relative to the equivalent normoxic circumstances and are the average (±SD) of two separate, independent tests carried out in duplicate. CHX: Cycloheximide

Supplementary Methods – LC–MS/MS parameters:

Samples (10 µl of peptides) were pre-concentrated on a C18 trap column (Acclaim PepMap RSLC, Thermo Fisher Scientific, MA, USA) for 10 minutes at 5 µl/min, then separated on a 50 cm C18 analytical column (75 µm ID, 2 µm particle size, 100 Å). Chromatographic separation was carried out on an RSLC-nano HPLC system using a linear gradient of 4–40% solvent B (80% acetonitrile in 0.1% formic acid) over 110 minutes at 300 nl/min. The column temperature was maintained at 35 °C.
